# Matrine inhibits IL-1β secretion in primary porcine alveolar macrophages through the MyD88/NF-κB pathway and NLRP3 inflammasome

**DOI:** 10.1186/s13567-019-0671-x

**Published:** 2019-07-12

**Authors:** Panpan Sun, Na Sun, Wei Yin, Yaogui Sun, Kuohai Fan, Jianhua Guo, Ajab Khan, Yongming He, Hongquan Li

**Affiliations:** 1 College of Animal Science and Veterinary Medicine, Shanxi Agricultural University, Taigu, 030801 Shanxi People’s Republic of China; 2 School of Life Science and Engineering, Foshan University, Foshan, 528000 Guangdong People’s Republic of China; 3 Laboratory Animal Center, Shanxi Agricultural University, Taigu, 030801 Shanxi People’s Republic of China; 4 Department of Veterinary Pathobiology, Schubot Exotic Bird Health Center, Texas A&M University, College Station, TX 77843 USA

## Abstract

Our previous studies demonstrated that matrine directly acts on the replication process of porcine reproductive and respiratory syndrome virus (PRRSV). Matrine inhibits viral replication and is also associated with the NF-κB signalling pathway. These results suggest that matrine has antiviral and anti-inflammatory effects. However, the specific anti-inflammatory mechanism of matrine is still unclear. In this study, we investigated the anti-IL-1β mechanism of matrine, as IL-1β is a major inflammatory cytokine, in porcine alveolar macrophages (PAMs) stimulated with 4 μg PRRSV 5′-untranslated region (UTR) RNA and 1 μg/mL LPS. After 5′UTR RNA and LPS co-stimulation of PAMs for 12 h, the expression of IL-1β, IL-6, IL-8 and TNF-α was significantly increased. The results also showed that co-stimulation induced the expression of MyD88, and activated the NF-κB signalling pathway and NLRP3 inflammasome. Furthermore, matrine treatment downregulated MyD88, NLRP3 and caspase-1 expression, inhibited ASC speck formation, suppressed IκBα phosphorylation, and interfered with the translocation of NF-κB from the cytoplasm to the nucleus. These results suggest that matrine plays an important role in PAMs co-stimulated with PRRSV 5′UTR RNA and LPS via its effect on NF-κB and the NLRP3 inflammasome. These findings lay the foundation for the exploration of the clinical application of matrine in PRRSV disease.

## Introduction

IL-1β, a potent pro-inflammatory cytokine, predominantly produced by macrophages, monocytes and dendritic cells, that plays a central role in inflammatory and immune response regulation [1]. Biologically, active IL-1β production and secretion involves at least two signalling cascades [2–4]. In the first cascade, pattern recognition receptors (PRRs) in host cells detect microorganisms and induce the transcription of an inactive 31-kDa precursor, termed pro-IL-1β. The Toll-like receptors (TLRs) are a family of PRRs, which once activated by pathogen-associated molecular patterns (PAMPs), potentiate NF-κB transcription pathway-related signalling cascades, ultimately causing the upregulation of pro-inflammatory cytokines [1, 3]. In the second signalling cascade, a large multimeric protein complex known as the NLRP3 inflammasome, consisting of NLRP3, apoptosis-associated speck-like protein (ASC) and pro-caspase-1, is formed. NLRP3 protein recruits ASC, which interacts with pro-caspase-1, promoting its cleavage and activation. Once activated, caspase-1 elevates pro-IL-1β processing to its biologically active form [5–7].

Infection by pathogens, including viruses, either up- or down-regulates cytokine levels. Previous studies have demonstrated that porcine reproductive and respiratory syndrome virus (PRRSV) infection induces interstitial pneumonia; the production of various pro-inflammatory cytokines, including IL-1β, IL-6, IL-8, and TNF-α, is significantly increased during PRRSV infection and correlates with persistent infection and the tissue pathology associated with PRRSV [8–11]. Currently, there are increasing numbers of studies on the mechanism of PRRSV initiating the host inflammatory response. Highly pathogenic PRRSV infection enhances IL-1β secretion that is dependent on the TLR4/MyD88/NF-κB/MAPK pathway and on the activation of NLRP3 inflammasome [12, 13]. The PRRSV nucleocapsid (N) protein also interacts with DHX36, an upstream signal of MyD88, to activate NF-κB signalling [14]. PRRSV genomic RNA, and structural protein E is involved in the secretion of IL-1β [15, 16]. PRRSV is the primary pathogen, but co-infections commonly occur with bacterial or viral pathogens that aggravate the clinical symptoms in growing pigs [17–19]. Previous research has clearly demonstrated that PRRSV and bacterial lipopolysaccharide (LPS) act synergistically to multiply the inflammatory response of infected lungs or macrophages [17–19].

*Sophora flavescens* is an evergreen perennial shrub that grows mainly in Asia, and its roots have been used in traditional Chinese medicine for the treatment of asthma and inflammation [20]. Matrine, an alkaloid isolated from *Sophora flavescens Ait*, has been reported to exhibit a wide range of pharmacological effects, including anti-tumour, antioxidant, antiviral and anti-inflammatory effects [21–25]. In our previous work, we suggested that matrine exhibits antiviral activity against PRRSV and porcine circovirus (PCV2) [23, 24]; the underlying antiviral mechanisms of matrine may be mediated by partly regulating the TLR3/TLR4/NF-κB/TNF-α pathway [23]. Matrine treatment also improves pneumonia symptoms in PRRSV/PCV2 co-infected mice and attenuates inflammation in mice with acute LPS-induced lung injury induced (unpublished data), but the mechanism for this remains to be determined. Our previous study also showed that matrine directly inhibits the PRRSV replication process by inhibiting the activity of Nsp9 [26]. Matrine is known to have both antiviral and anti-inflammatory efficacy.

In this study, in order to avoid the interference of PRRSV infection and replication and to induce higher levels of IL-1β expression, we established an inflammatory model of porcine alveolar macrophages (PAMs) by transfecting PRRSV 5′UTR RNA and LPS. According to the IL-1β production and secretion process, we investigated the anti-IL-1β mechanism of matrine based on the inflammatory model.

## Materials and methods

### Cells, viruses and drugs

PAMs cultures were prepared as described in our previous studies [23]. In brief, PAMs were obtained from lung lavage fluid of healthy 4-week-old pigs with negative PCR and antibody test strip PRRSV and PCV2 results. Following 3 washes with PBS, the cells were frozen and stored in liquid nitrogen until use. The PAMs were maintained in 10% heat-inactivated foetal bovine serum (FBS) RPMI 1640 medium with 1% penicillin–streptomycin at 37 °C in an atmosphere with 5% CO_2_.

PRRSV strain JS-1 was kindly gifted by the Jiangsu Academy of Agricultural Sciences, China [27]. PRRSV was propagated in Marc-145 cells. Matrine (MT) was purchased from Nanjing Zelang Meditech Ltd., (Jiangsu, China) and its purity by HPLC was 98%. The concentration of matrine used in the experiments was based on our published results [23]. Dexamethasone (DEX) was purchased from Solarbio^®^ Life Sciences Ltd., (Beijing, China) and its purity was greater than 98%. The MTT (3-(4,5-dimethylthiazol-2-yl)-2,5-diphenyltetrazolium bromide) results showed that when PAMs were treated with 0.04 mg/mL dexamethasone, the cell survival rate was greater than 80%. In this study, dexamethasone was used as a positive anti-inflammatory control.

### Isolation of PRRSV RNA and preparation of 5′UTR transcripts

The PRRSV 5′UTR was amplified by PCR using specific primers listed in Table 1. PCR products were isolated by agarose gel, recovered with a gel extraction kit (TIAGEN, China) and inserted into the expression vector pcDNA3.1(+). Plasmids with the correct inserts were selected by colony PCR and confirmed by DNA sequencing (Beijing Genomics Institute, China). Plasmids were extracted and linearized with *Xho*I digestion (Takara, China) to serve as a template to synthesize the 5′UTR transcripts in vitro using the RiboMAX Large Scale RNA Production Systems (Promega, USA) according to the manufacturer’s instructions. After transcription, RNA was purified by a MEGAclear™ Purification Kit for Large Scale Transcription Reactions (Thermo Fisher, USA).

**Table 1:** **PCR primers used in this study**

Gene	Primer sequence
5′UTR	F: CGGGATCCATGACGTATAGGTGTTGGCT
	R: CCGCTCGAGGGTTAAAGGGGTGGAGAGA
IL-1β	F: CCCAAAAGTTACCCGAAGAGG
	R: TCTGCTTGAGAGGTGCTGATG
IL-6	F: ACAAAGCCACCACCCCTAAC
	R: CGTGGACGGCATCAATCTCA
IL-8	F: TTCACAAGTCTCTGCTCAACTG
	R: TGTCCTCAAGGTAGGATGGG
TNF-α	F: CCCTCACGTCCTTCTGGTTT
	R: GAGTCTGGAAGCCCCAGTTC
GAPDH	F: TTGGCTACAGCAACAGGGTG
	R: CAGGAGATGCTCGGTGTGTT

### Establishment of the PAMs inflammatory model

The frozen PAMs were thawed in a 37 °C water bath, adjusted to a density of 1 × 10^6^ cells/mL and seeded in 6-well plates. The medium was changed after a 2 h incubation. Following another 12 h incubation, the cells were transfected with various quantities of PRRSV 5′UTR RNA (1, 2, and 4 μg/well) along with 1 μg/mL LPS (RNA + LPS group). The cell control group, transfection reagents group (only the medium and transfection reagents, RNA-0), 5′UTR RNA group (only transfection with different doses of 5′UTR RNA, RNA-n) and LPS control group (only 1 μg/mL LPS added) was also prepared. The details of the experimental design are shown in Table 2. According to QIAGEN trans messenger transfection reagent (301525) instructions, the trans messenger-RNA transfection complexes were prepared by mixing RNA with enhancer R and buffer EC-R, incubation for 5 min at room temperature with an additional 10 min incubation after the addition of the trans messenger reagent. The complexes were mixed with medium (with LPS or without LPS) and added directly to the cells. After 12 h, PAMs from all groups were collected and used for the extraction of RNA to determine the mRNA expression levels of IL-1β, TNF-α, IL-8 and IL-6. In addition, the supernatant was collected for protein extraction to detect the levels of the corresponding inflammatory cytokines.

**Table 2:** **Experimental design for the induction of the inflammatory response in PAMs**

	Cell control group (Cell)	Transfection reagents group (RNA-0)	5′UTR RNA transfect group (RNA-n)	5′UTR RNA + LPS group (RNA-n + LPS)	LPS control group (LPS)
Transfection reagents	−	+	+	+	−
5′UTR RNA	−	−	+	+	−
1 μg/mL LPS	−	−	−	+	+

n represents the RNA dose used for transfection, *n* = 1, 2 or 4 μg.

### Effects of matrine on the inflammatory response of PAMs

PAMs were prepared as described in the section on the Establishment of the PAMs inflammatory model. The experimental design is shown in Table 3. Seven groups were included in this assay: cell control group, transfection reagents group, 5′UTR RNA and LPS co-stimulation group, matrine treatment group (0.4, 0.2 and 0.1 mg/mL) and dexamethasone treatment group (0.04 mg/mL). At 6 h post-transfection, the old medium in the wells was removed, and the appropriate concentration of matrine or dexamethasone prepared in fresh medium was added to the plate. An equal volume of medium was added to the control cells. After another 6 h incubation, PAMs and supernatants were collected for RNA and protein detection.

**Table 3:** **Experimental design for the assessment of the effects of matrine on the PAMs inflammatory response**

	Cell control group (Cell)	Transfection reagents group (RNA-0)	5′UTR RNA + LPS group (RNA-4 + LPS)	Matrine treatment group (MT)	Dexamethasone treatment group (DEX)
Transfection reagents	−	+	+	+	+
4 μg 5′UTR RNA	−	−	+	+	+
1 μg/mL LPS	−	−	+	+	+
Matrine	−	−	−	+	−
Dexamethasone	−	−	−	−	+

At 6 h post-transfection, the transfected medium was removed in the matrine or dexamethasone treatment groups. Fresh medium with 0.4 mg/mL matrine was added to the matrine treatment group, and fresh medium with 0.04 mg/mL dexamethasone was added to the dexamethasone treatment group.

### qRT-PCR

Total RNA from 2 × 10^6^ cells was extracted with TRIzol Reagent (Takara, China), and the RNA concentrations were determined using a NanoDrop 1000 spectrophotometer (NanoDrop Technologies, USA). cDNA synthesis was performed according to the Prime Script™ RT Reagent Kit with gDNA Eraser manufacturer’s protocol (Takara, China).

Using the SYBR Green detection system (Biotool, China), gene expression levels of IL-1β, TNF-α, IL-8 and IL-6 were detected by qRT-PCR (Applied Biosystems^®^ 7500 Real-Time PCR system). The gene expression was normalized to that of glyceraldehyde-3-phosphate dehydrogenase (GAPDH). The relative expression of genes was analysed using the 2^−∆∆Ct^ method. The primer sequences for IL-1β, TNF-α, IL-8, IL-6 and GAPDH are shown in Table 1.

### Protein extraction and Western blot analysis

PAMs were washed twice with pre-chilled PBS after the medium was removed. Total cell extracts were prepared with a cell lysis buffer (Beyotime Biotechnology, China) or a nuclear protein and cytosolic protein extraction kit (KeyGene Biotech, China) supplemented with protease inhibitors. The supernatants were collected and stored at −80 °C, and the protein concentration was determined using a bicinchoninic acid protein assay reagent (Beyotime Biotechnology, China).

The supernatant protein was centrifuged at 10 000 × *g* for 10 min at 4 °C after the supernatant was mixed with a fourfold volume of pre-cooled acetone and incubated at −80 °C overnight. After the supernatant was discarded with caution, the pellet was allowed to dry at room temperature for 30 min. A total of 40 μL of 1× SDS loading buffer was added to dissolve the protein pellet.

The proteins were resolved by 10–15% SDS-PAGE and transferred to a polyvinylidene difluoride membrane (Millipore, USA). The membrane was blocked with 5% non-fat milk for 2 h and then incubated with different antibodies (Table 4) for 2 h at room temperature or overnight at 4 °C. After washing three times with TBST (TBS with 0.5% Tween 20), the membrane was incubated with an HRP-conjugated secondary antibody for 1 h at 37 °C (Table 5). Following another three washes with TBST, the protein bands were detected with an ECL Western Blot Kit (CWbio Inc., China). GAPDH was used as an internal control for the normalization of all proteins except nuclear p65, and TATA-binding protein (TBP) was used to normalize the expression of nuclear proteins.

**Table 4:** **The details of the primary antibodies used in this study**

Name	Host	Dilution ratio	Cat. no.	Manufacturer
IL-1 β	Rabbit	1:1000	ASC0912	Thermo Fisher, USA
IL-6	Goat	1:500	PP690	
IL-8	Goat	1:600	AF535	R&D Systems, USA
TNF-α	Goat	1:600	AF690	
p-IκBα	Mouse	1:500	9246	CST, USA
TLR4	Rabbit	1:500	19811-1-AP	Proteintech, Wuhan
MyD88	Rabbit	1:500	23230-1-AP	
p65	Rabbit	1:500	10745-1-AP	
IκBα	Rabbit	1:500	10268-1-AP	
TBP	Mouse	1:500	66166-1-lg	
GAPDH	Mouse	1:5000	60004-1-lg	
NLRP3	Rabbit	1:500	/	Kindly gift by Prof. Weng, Harbin Veterinary Research Institute of Chinese Academy of Agricultural Sciences
Caspase-1	Rabbit	1:1000	/	
ASC	Mouse	1:500	/	
ASC	Rabbit	1:200	bs-6741R	Bioss, Beijing

**Table 5:** **The details of the secondary antibodies used in this study**

Name	Dilution ratio	Cat. no.	Manufacturer
Goat anti-rabbit	1:20 000	CW0103S	Comwin Biotech, Beijing
Goat anti-mouse	1:20 000	CW0102S	
Rabbit anti-goat	1:20 000	CW0105S	
Alexa fluor 488-conjugated goat anti-rabbit IgG	1:200	SA0006-2	Proteintech, Wuhan

### Immunofluorescence confocal microscopy

The cells were immobilized with pre-cooled 80% acetone. ASC was detected using a primary rat mAb and secondary Alexa Fluor 488-conjugated goat anti-rat IgG antibody. Nuclei were stained with DAPI (KeyGEN BioTECH, China). The stained cells were examined using a laser scanning microscope (Leica sp80, Germany).

### Statistical analysis

The experiments were repeated three times with three replicates within each experiment. The results are presented as the mean ± standard error of the mean (SEM). The grey scale Western blot images were analysed by ImageJ software [28]. The statistical significance of the differences between parameters was evaluated via one-way analysis of variance (ANOVA) by GraphPad Prism 5 software.

## Results

### PRRSV 5′UTR RNA and LPS co-stimulation induced the secretion of inflammatory mediators

**Figure 1:**
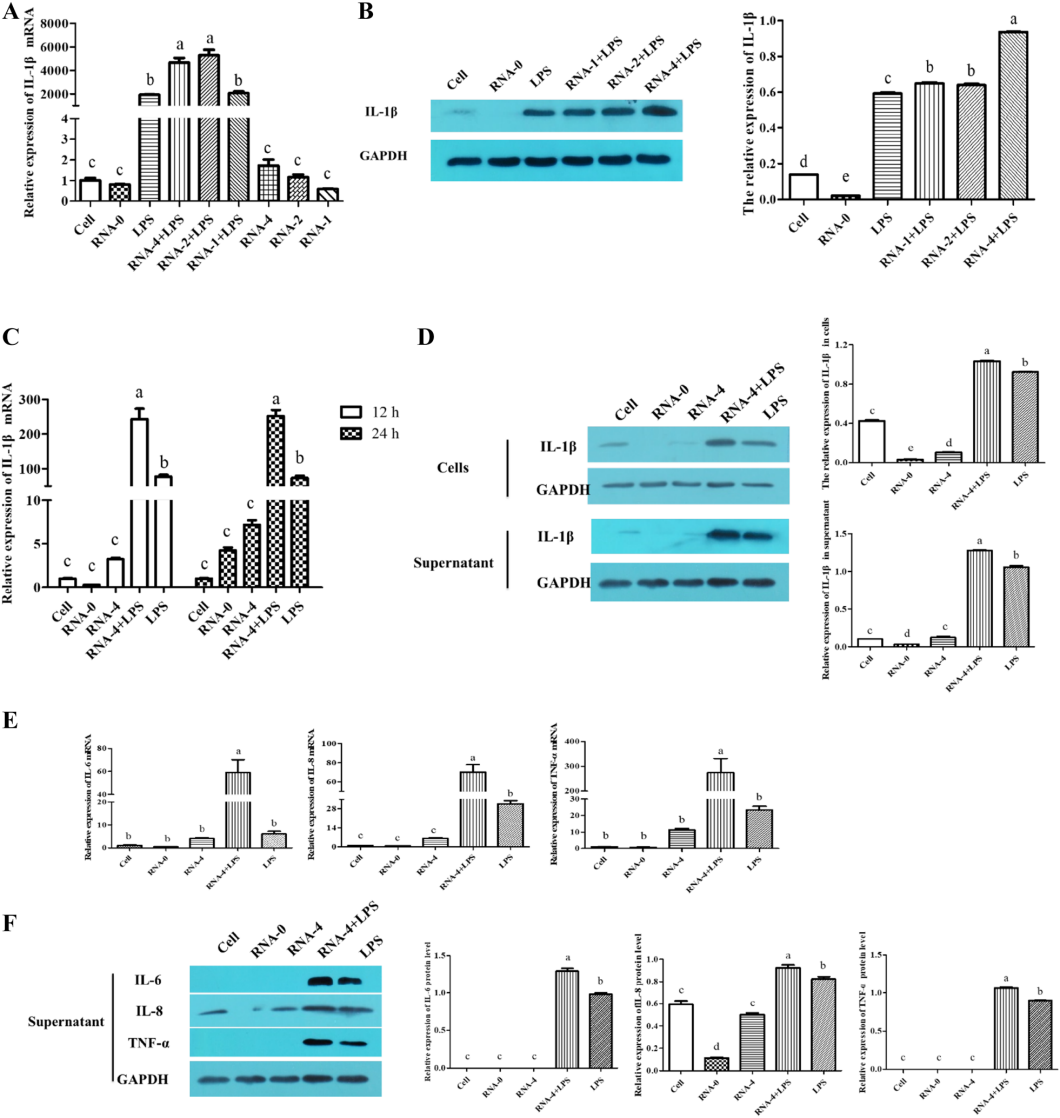
**Inflammatory response in PAMs induced by PRRSV 5′UTR RNA and LPS. A**, **B** PAMs were transfected with different doses of 5′UTR RNA (1, 2, and 4 μg/well) along with 1 μg/mL LPS. qRT-PCR and Western blot results showed that compared with PAMs in other groups, PAMs in the 4 μg 5′UTR RNA and 1 μg/mL LPS co-stimulation group produced higher levels of IL-1β (*p* < 0.05). **C** After co-stimulation, the relative expression level of IL-1β mRNA obtained was similar at the 12 and 24 h time-points. **D** 5′UTR RNA and LPS co-stimulation induced IL-1β expression in cells and supernatants. **E**, **F** 5′UTR RNA and LPS co-stimulation induced increased levels of IL-6, IL-8 and TNF-α mRNA and protein. Expression was normalized to that of GAPDH. Different letters (a, b, c, d, and e) on data indicate significant differences between groups (*p* < 0.05).

The expression of inflammatory mediators, including IL-1β, IL-6, IL-8 and TNF-α, was measured in PAMs treated with 5′UTR RNA alone, LPS alone or 5′UTR RNA and LPS in combination. The results showed that the IL-1β mRNA expression level was much higher in the 5′UTR RNA and LPS co-stimulation group than in the 5′UTR RNA alone and LPS alone groups (*p* < 0.05) (Figure 1A). The mRNA levels of IL-1β in the 4 and 2 μg co-stimulation groups were significantly higher than those in the 1 μg co-stimulation group (*p* < 0.05). The Western blot results also indicated that the IL-1β protein expression level in the 4 μg RNA and LPS co-stimulation group was significantly higher than that of the other groups (Figure 1B). As shown in Figure 1C, after co-stimulation with 5′UTR RNA and LPS for 12 h and 24 h, the level of IL-1β mRNA at these two time-points was similar. The expression of IL-1β protein was also detected in whole cell lysates and in supernatants (Figure 1D). The results showed that compared with other groups, the 4 μg 5′UTR RNA and LPS group had the highest IL-1β expression level (*p* < 0.05). Therefore, in the subsequent mechanistic studies of matrine, PAMs were incubated for 12 h after co-stimulation with 4 μg 5′UTR RNA and LPS.

In addition to IL-1β expression, we also measured the IL-6, IL-8 and TNF-α expression after 12 h of stimulation. As shown in Figures 1E and F, the mRNA and protein levels of these inflammatory cytokines in the supernatants were much higher in the 5′UTR RNA and LPS co-stimulation group than in the other control groups (*p* < 0.05).

### Matrine inhibited the secretion of inflammatory mediators

**Figure 2:**
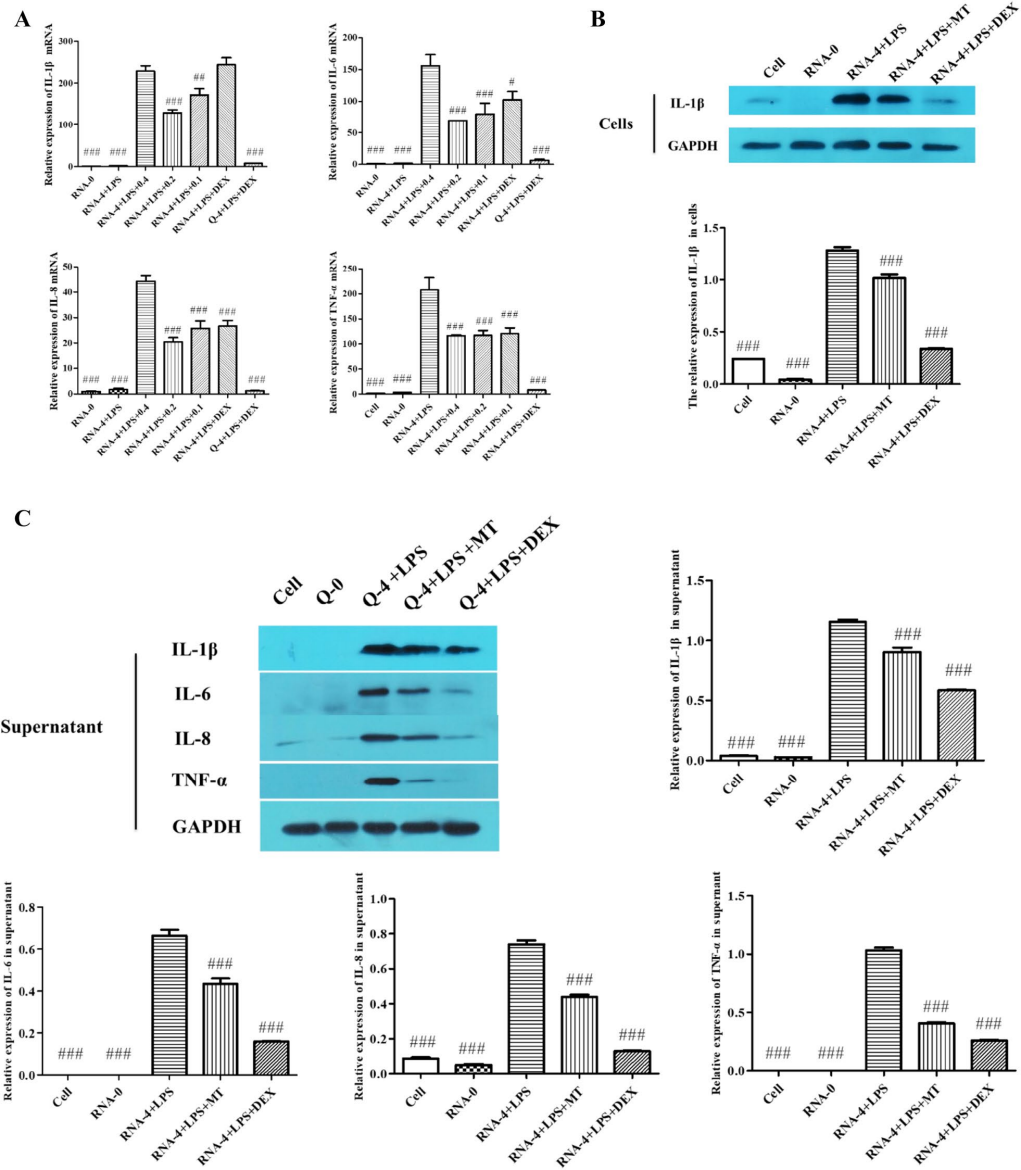
**Matrine inhibited the secretion of inflammatory mediators. A** Matrine inhibited the mRNA expression of IL-1β, IL-6, IL-8 and TNF-α. After 6 h of co-stimulation, different doses of matrine (0.4, 0.2 and 0.1 mg/mL) or 0.04 mg/mL dexamethasone were added to the cells for an additional 6 h of incubation. The expression levels of IL-1β, IL-6, IL-8 and TNF-α genes in PAMs from all treatment groups were measured using qRT-PCR. **B**, **C** Matrine reduced the protein levels of IL-1β, IL-6, IL-8 and TNF-α. Expression was normalized to GAPDH expression. ^#^Indicates that the expression was significantly different compared to that of the group with 5′UTR RNA and LPS co-stimulation (*p* < 0.05).

Macrophages play a key role during inflammatory response initiation and exert pro-inflammatory effects by secreting cytokines, such as IL-1β, IL-6, IL-8 and TNF-α. Therefore, we investigated the effects of matrine on the secretion of these inflammatory mediators to evaluate its anti-inflammatory effect. As shown in Figure 2A, when compared with that of the co-stimulation group, the expression of IL-1β was significantly decreased in the 0.4 and 0.2 mg/mL matrine treatment groups (*p* < 0.05), and the 0.1 mg/mL matrine group showed no significant difference in IL-1β mRNA expression (*p* > 0.05). For IL-6, IL-8 and TNF-α, all matrine dose groups showed significantly decreased mRNA expression levels (*p* < 0.05). All of these results indicate that matrine treatment inhibited the expression of inflammatory cytokines in PAMs co-stimulated with 5′UTR RNA and LPS. Matrine inhibited the expression of IL-1β and IL-6 in a dose-dependent manner.

At the protein level, the 0.4 mg/mL matrine group showed significantly inhibited IL-1β expression when compared with the 5′UTR RNA and LPS co-stimulation group (*p* < 0.05) (Figure 2B). The protein bands for IL-1β, IL-6, IL-8 and TNF-α were detected in the cell supernatant. Compared with the co-stimulation group, the matrine-treated group showed significantly inhibited IL-1β, IL-6, IL-8 and TNF-α expression (*p* < 0.05) (Figure 2C).

### Matrine treatment blocked NF-κB activation

**Figure 3:**
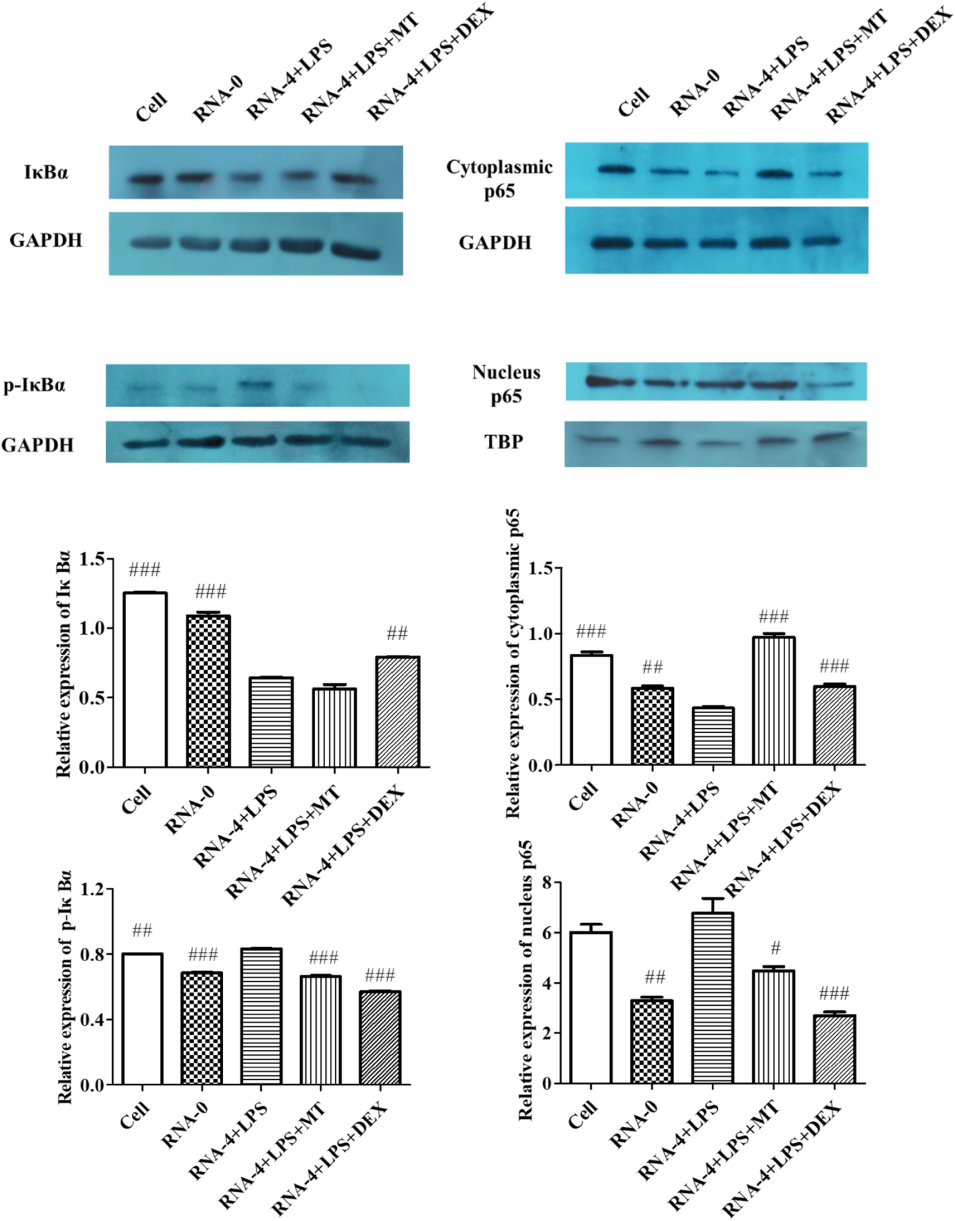
**Matrine inhibited NF-κB activation.** The protein levels of IκBα, p-IκBα, cytoplasmic p65 and nuclear p65 were measured by Western blot assay (upper), and GAPDH was used as an internal control for normalization. The expression of nuclear p65 was normalized to that of TBP using ImageJ software (lower). ^#^Indicates that the expression was significantly different compared to that of the group with 5′UTR RNA and LPS co-stimulation (*p* < 0.05).

NF-κB plays a critical role in regulating the inflammatory response and production of various pro-inflammatory mediators. The degradation and phosphorylation of IκBα and nuclear translocation of p65 are important events in NF-κB signalling activation. To investigate how matrine modulates the NF-κB signalling pathway in PAMs after 5′UTR RNA and LPS co-stimulation, Western blotting was used to detect NF-κB p65, IκBα, and phospho-IκBα levels. Figure 3 shows that compared with the cell control and transfection reagent control groups, the 5′UTR RNA and LPS co-stimulation group showed significantly decreased protein levels of IκBα and cytoplasmic p65, and increased levels of p-IκBα and nucleus p65. This result indicates that 5′UTR RNA and LPS co-stimulation induced the translocation of NF-κB from the cytoplasm to the nucleus, which further indicates that the NF-κB pathway was activated. The expression of p-IκBα and p65 (both in the cytoplasm and nucleus), but not IκBα expression, was significantly different between the co-stimulation group and the matrine treatment group, demonstrating that matrine treatment triggered p-IκBα degradation and the nuclear translocation of p65, which inhibited NF-κB activation.

### Matrine inhibited the NF-κB signalling pathway through MyD88

**Figure 4:**
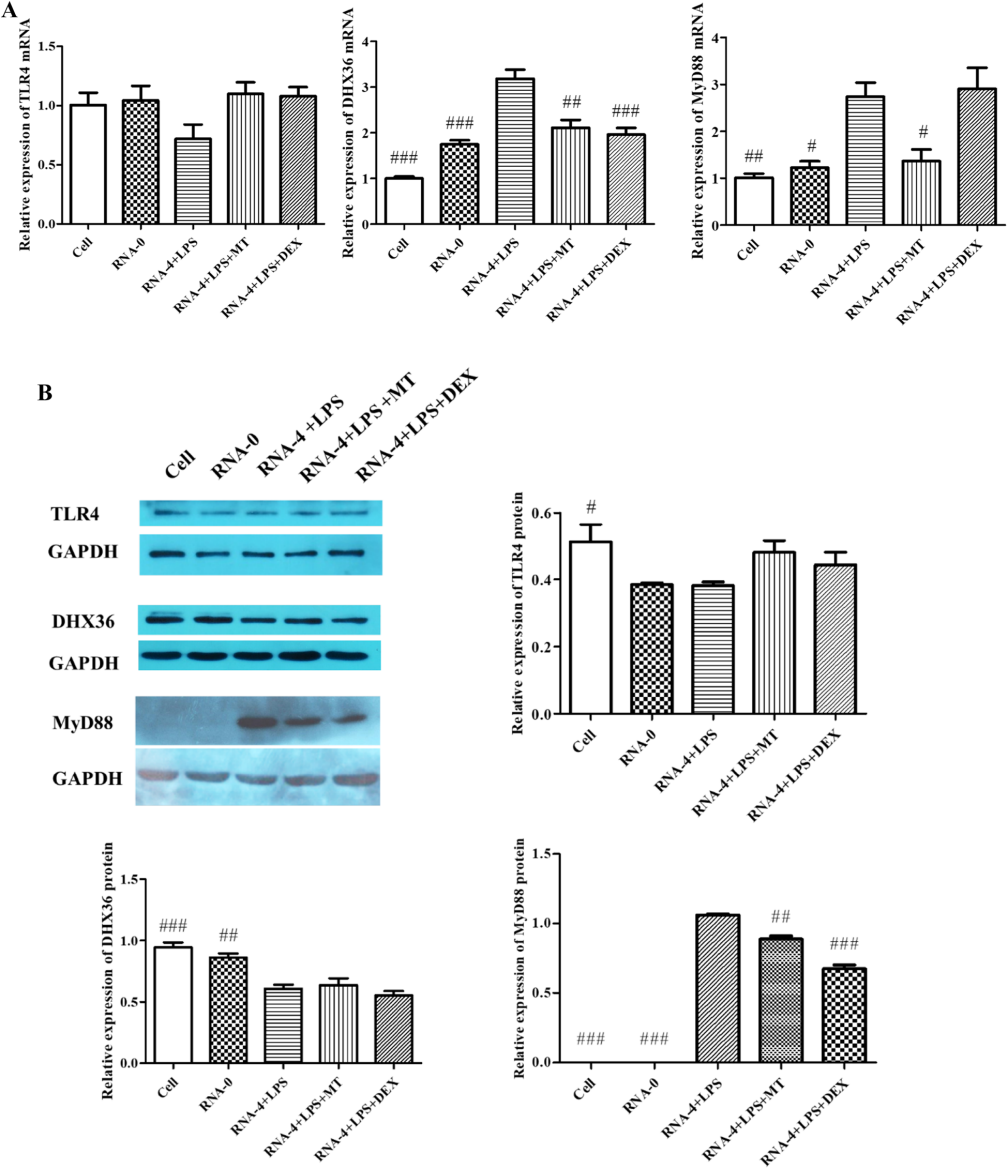
**Effect of matrine on TLR4, DHX36 and MyD88 expression.** PAMs from all treatment groups were collected, and total RNA or protein was extracted and assessed by qRT-PCR or Western blot, respectively. **A** The expression of the TLR4, DHX36 and MyD88 genes. The results showed no changes in TLR4 gene expression levels among the different groups (*p* > 0.05), but DHX36 and MyD88 gene expression levels were significantly increased in the 5′UTR RNA and LPS co-stimulation group (*p* < 0.05). Compared with the co-stimulation group, the matrine-treated group showed reduced expression of DHX36 and MyD88 genes (*p* < 0.05). **B** The expression of TLR4, DHX36 and MyD88 protein levels. Expression was normalized to that of GAPDH. ^#^Indicates that the expression was significantly different compared to that of the group with 5′UTR RNA and LPS co-stimulation (*p* < 0.05).

As shown in Figure 4A, compared with the cell and transfection reagent control groups, the 5′UTR RNA and LPS co-stimulation group showed increased mRNA expression of DHX36 and MyD88 (*p* < 0.05), but not TLR4 (*p* > 0.05). Compared with the 5′UTR RNA and LPS co-stimulation group, the matrine treatment group showed significantly decreased expression of DHX36 and MyD88 (*p* < 0.05). At the protein level (Figure 4B), 5′UTR RNA and LPS co-stimulation significantly increased MyD88 expression when compared with that of the transfection reagent control (*p* < 0.05), but decreased the expression of DHX36 (*p* < 0.05), and had no effect on TLR4 expression (*p* > 0.05). In the matrine treatment group, MyD88 expression was significantly decreased compared with that in the 5′UTR RNA and LPS co-stimulation group (*p* < 0.05); however, there were no significant differences in TLR4 and DHX36 expression (*p* > 0.05). These results indicate that matrine inhibited the expression of MyD88 and decreased the expression of DHX36 at the mRNA level.

### Matrine reduced the activation of the NLRP3 inflammasome

**Figure 5:**
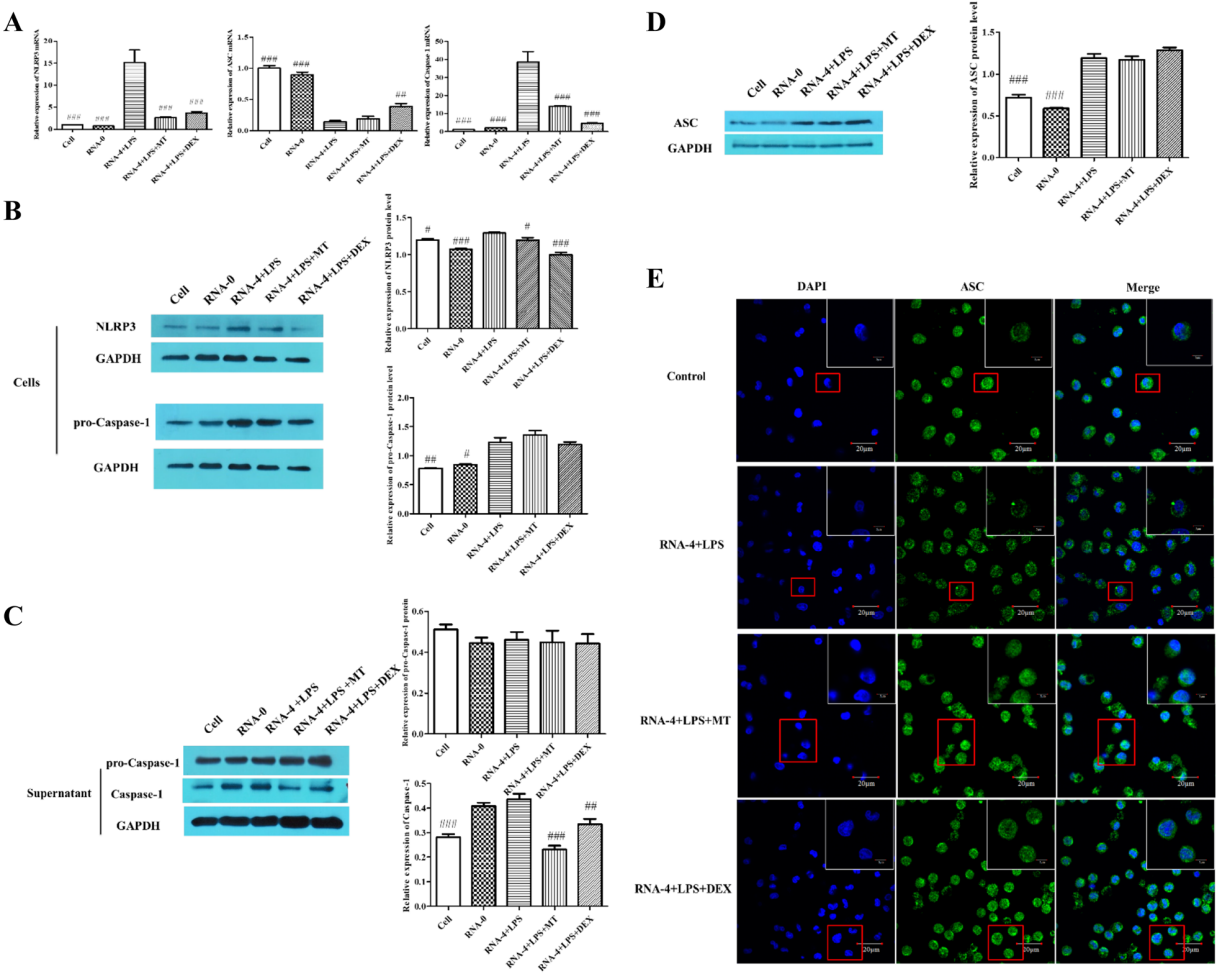
**Matrine inhibited NLRP3 inflammasome activation in PAMs. A** The mRNA expression of NLRP3, ASC and caspase-1 was detected by qRT-PCR. **B**, **C** Protein levels in PAMs were assessed with antibodies against NLRP3 and caspase-1 by immunoblotting (left panel). Quantification of the protein expression was performed by densitometric analysis of the bands (right panel). **D** Effects of matrine on ASC protein expression. Expression was normalized to GAPDH expression. **E** ASC specks (FITC) and nuclei (DAPI) were visualized using a confocal fluorescence microscope. ASC is evenly distributed in the cytoplasm and nucleus (control), and 5′UTR RNA and LPS co-stimulation induced the formation of ASC specks near the nuclear membrane. Matrine inhibited the formation of ASC specks. ^#^Indicates that the expression was significantly different compared to that of the group with 5′UTR RNA and LPS co-stimulation (*p* < 0.05).

The most intensively studied inflammasome complex is NLRP3. Once activated, NLRP3 directly interacts with ASC, and then ASC interacts directly with pro-caspase-1. NLRP3 inflammasome formation activates caspase-1 and the production of mature IL-1β. In this study, after 12 h of transfection, NLRP3, ASC and caspase-1 gene/protein levels in PAMs from all treatment groups were measured using qRT-PCR and Western blot analysis. Figures 5A and B show that the expression of NLRP3 and caspase-1 in the 5′UTR RNA and LPS co-stimulation group was much higher than that in the cell and transfection reagent control groups (*p* < 0.05). Compared with the 5′UTR RNA and LPS co-stimulation alone, matrine treatment decreased the expression of NLRP3 and the mRNA level of caspase-1 (*p* < 0.05) but had no discernible effect on the protein expression level of pro-caspase-1 (*p* > 0.05). In supernatant protein (Figure 5C), when compared with the cell control, 5′UTR RNA and LPS co-stimulation significantly increased the level of caspase-1 (*p* < 0.05) but had no effect on pro-caspase-1 secretion (*p* > 0.05). Compared with the 5′UTR RNA and LPS co-stimulation group, the matrine treatment group showed significantly decrease expression of caspase-1 (*p* < 0.05).

For ASC (Figures 5A and D), the 5′UTR RNA and LPS co-stimulation group showed significantly decreased ASC mRNA expression but increased protein expression of ASC when compared with that of the control groups (*p* < 0.05). When compared with the 5′UTR RNA and LPS co-stimulation group, the matrine treatment group showed no difference in the expression of ASC at either the gene or protein level (*p* > 0.05). ASC forms large aggregates called “specks” when the inflammasome is activated. Therefore, we subsequently tested whether matrine influenced the formation of ASC speck. As shown in Figure 5E, ASC was evenly distributed in the cytoplasm and nucleus of normal cells, while 5′UTR RNA and LPS co-stimulation induced the formation of ASC specks near the nuclear membrane. In contrast, matrine treatment inhibited the formation of ASC specks. Taken together, these results show that matrine inhibits NLRP3 inflammasome activation.

## Discussion

Natural products and their derivatives exhibit antiviral and anti-inflammatory effects. Matrine, like other natural products, possesses effective anti-inflammatory activity in mice with acute lung injury [29] and arthritis [21], and also exhibits antiviral activity. Our previous study indicated that the antiviral mechanisms of matrine may be mediated partly by regulating the TLR3, 4/NF-κB/TNF-α pathway [23]. However, the anti-inflammatory molecular mechanisms of matrine are poorly understood.

One of the most important pro-inflammatory cytokines, IL-1β, is produced mainly by monocytes and macrophages. Upon virus infection and subsequent inflammatory responses, IL-1β exhibits a broad range of biological effects, including activation of the innate immune system and modulation of the adaptive immune response [1, 2]. The generation of IL-1β requires two steps: IL-1β is synthesized as an inactive precursor protein in the first regulatory step, involving the TLR/MyD88/NF-κB signal pathway; this precursor is cleaved intracellularly by caspase-1 (IL-1β convertase) to form the active form of the protein that is later secreted in the second step, activating the NLRP3 inflammasome [1, 3, 5, 6].

The most prominent clinical sign of PRRSV infection is interstitial pneumonia in growing pigs, which suggests that the inflammatory response plays an important role in PRRSV pathogenesis. The levels of various pro-inflammatory cytokines, including IL-1β, IL-6, IL-8, and TNF-α, are increased significantly during PRRSV infection, and correlate with persistent infection and the tissue pathology associated with PRRSV [8–11]. Previous studies have shown that in PAMs, PRRSV induces IL-1β production dependent on the TLR4/MyD88 pathway and NLRP3 inflammasome [12]. DexD/h-Box helicase 36 (DHX36)-MyD88 plays a relevant role in the recognition of PRRSV nucleocapsid protein and in the subsequent activation of the pro-inflammatory NF-κB pathway [14]. Li et al. found that a member of the DEAD/H-box protein family called DDX19A senses PRRSV RNA and mediates NLRP3-dependent inflammasome activation to affect pro-caspase-1 cleavage and IL-1β secretion in PAMs [15]. In the induction of multifactorial respiratory diseases, the synergistic effects of PRRSV and a secondary bacterial infection are being recognized, and bacterial and viral co-infection can induce a more severe inflammatory response than single infections [17–19].

In the present study, PAMs transfected with 4 μg PRRSV 5′UTR RNA and treated with 1 μg/mL LPS to induce high levels of IL-1β were used as the inflammatory model to study the specific anti-IL-1β mechanism of matrine. The obtained data are consistent with the expectation that co-stimulating PAMs with PRRSV 5′UTR RNA and LPS induces higher expression of pro-inflammatory factors (IL-1β, IL-6, IL-8 and TNF-α) than stimulation with PRRSV 5′UTR RNA or LPS alone. The expression and secretion of IL-1β, IL-6, IL-8 and TNF-α was strongly inhibited by matrine. These observations are consistent with previous studies showing that matrine has an anti-inflammatory function [21, 25, 29].

TLRs play a critical role in innate immune responses by recognizing distinct PAMPs. Once activated, TLRs recruit the intracellular adaptor protein MyD88 to the Toll-IL-1-receptor (TIR) domain; subsequently, IRAKs and IKKβ are phosphorylated, resulting in the translocation of NF-κB to the nucleus [2]. In this study, we examined how matrine affects the TLR4/MyD88/NF-κB pathway after co-stimulation with PRRSV 5′UTR RNA and LPS. Our results demonstrated that PRRSV 5′UTR RNA and LPS co-stimulation induces IκBα degradation and phosphorylation as well as the translocation of NF-κB from the cytoplasm to the nucleus. In addition, PRRSV 5′UTR RNA and LPS co-stimulation affected the expression of MyD88 but not TLR4. This may be because PRRSV 5′UTR RNA was introduced into cells by transfection, which could not have an effect on cell membrane surface receptor expression. DHX36, an upstream signal of MyD88, interacts with PRRSV N proteins to activate NF-κB signalling [14]. In this study, PRRSV 5′UTR RNA and LPS co-stimulation induced the mRNA expression of DHX36 but decreased the protein expression of DHX36. The exact cause is unknown but is probably because PRRSV 5′UTR RNA is not a direct DHX36 target. Moreover, we found that matrine suppressed MyD88, p-IκBα and p65 expression in the nucleus and increased cytoplasmic p65 expression, suggesting that matrine blocks the activation of NF-κB. Previous studies have revealed that many anti-inflammatory drugs affect the TLR4/MyD88/NF-κB pathway. Chung et al. [30] demonstrated that Shinbaro3 modulates the TLR4/MyD88 pathway to exert anti-inflammatory effects in LPS-stimulated RAW 264.7 macrophage cells. Jia et al. [31] reported that berbamine exerts anti-inflammatory effects via the inhibition of the NF-κB and MAPK signalling pathways. Wang et al. [32] demonstrated that phenolics, extracted from Jujube peel, exert anti-inflammatory effects in LPS-stimulated murine macrophages through the MAPK and NF-κB pathways.

NF-κB signalling controls pro-IL-1β expression. The NLRP3 inflammasome complex, composed of NLRP3, ASC and caspase-1, is activated by PAMPs or danger-associated molecular patterns (DAMPs), resulting in the activation of caspase-1 and cleavage of pro-IL-1β to form active IL-1β; the NLRP3 inflammasome can cause serious inflammatory conditions when excessively activated [5, 6, 33]. Previous studies reported that TLR ligands or cytokines could prime NLRP3 through the NF-κB signalling pathway and that other stimuli, such as ATP, nigericin, or particulate matter, are necessary for NLRP3 activation [4, 6, 33]. Cao et al. reported that naringin, extracted from the most abundant flavonoid grapefruit, inhibits DSS-induced ulcerative colitis through NF-κB, MAPK and the NLRP3 inflammasome [34]. Zhao et al. noted that magnesium isoglycyrrhizinate blocks fructose-induced hepatic NF-κB/NLRP3 inflammasome activation [35]. Liu et al. indicated that the inflammatory response in asthmatic mice was attenuated by Yupingfeng San, which inhibits the NLRP3 inflammasome [36]. In this study, our results indicated that the expression of NLRP3, ASC and caspase-1 was increased and speck formation was also detected in the PRRSV 5′UTR RNA and LPS co-stimulation groups. Matrine attenuated NLRP3 activation induced by PRRSV 5′UTR RNA and LPS, suppressed the expression of NLRP3 and caspase-1, and suppressed ASC speck formation.

**Figure 6:**
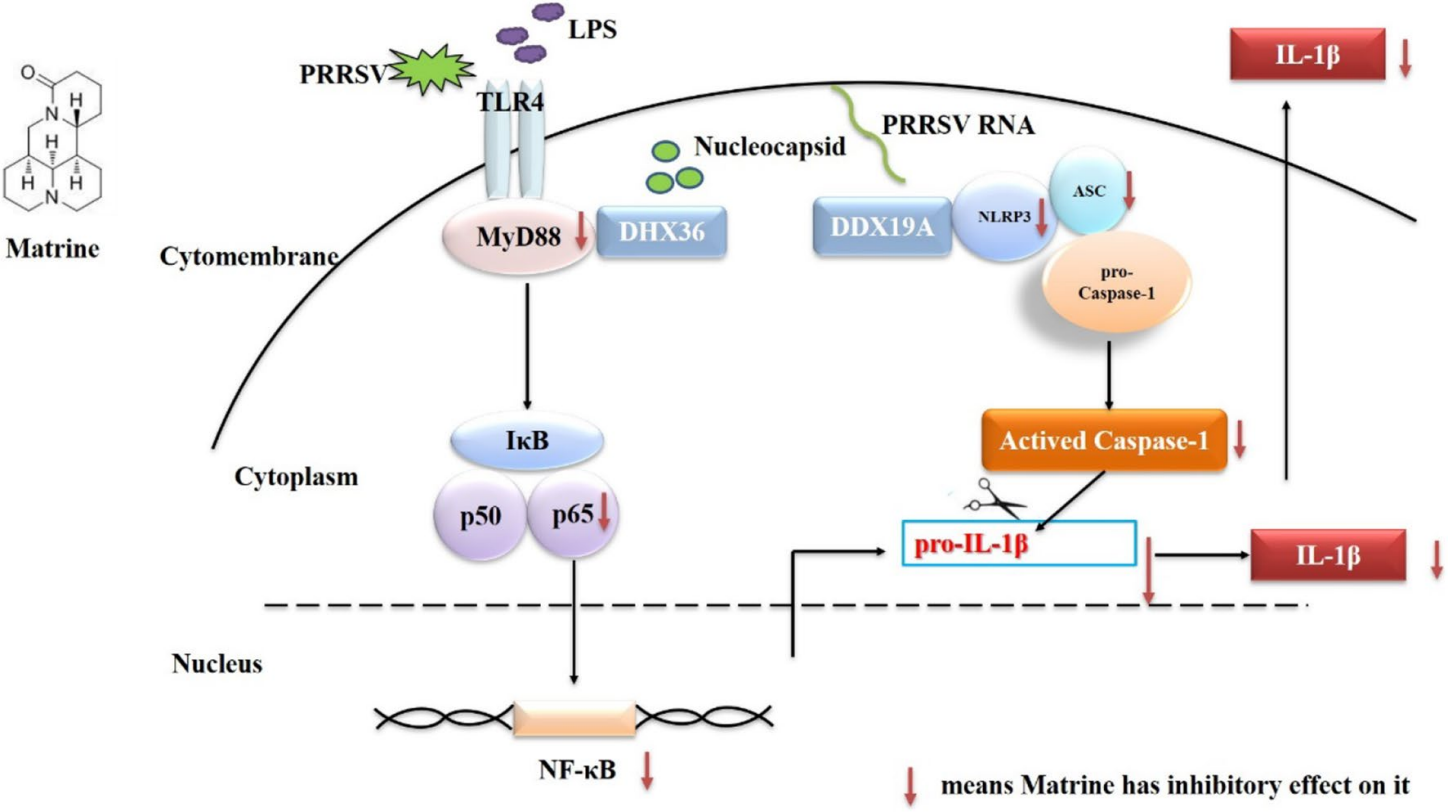
**Proposed model illustrating the anti-IL-1β activity of matrine.** 5′UTR RNA and LPS co-stimulation induces the recruitment of MyD88 to activate the NF-κB signalling pathway, which in turn initiates NF-κB-dependent pro-IL-1β, IL-6, IL-8 and TNF-α transcription and also induces the activation of the NLRP3 inflammasome. Matrine treatment downregulates MyD88, NLRP3 and caspase-1 expression, inhibits ASC speck formation, suppresses IκBα phosphorylation, and interferes with the translocation of NF-κB from the cytoplasm to the nucleus.

PRRSV infection leads to the increased release of IL-1β both in vitro and in vivo [8, 9, 11–13]. We found that matrine inhibited IL-1β production by inhibiting MyD88/NF-κB and NLRP3 inflammasome activation in vitro (Figure 6). Therefore, matrine might be used to inhibit inflammatory response in pigs infected by PRRSV. Our ongoing research will validate the anti-inflammatory mechanism of matrine in vivo and explore the other functions of matrine.

## Abbreviations

PRRSV: porcine reproductive and respiratory syndrome virus
PAMs: porcine alveolar macrophages
UTR: untranslated region
PRRs: pattern recognition receptors
TLRs: Toll-like receptors
MyD88: myeloid differentiation primary response 88
PAMPs: pathogen-associated molecular patterns
ASC: apoptosis-associated speck-like protein
DHX36: DexD/h-Box helicase 36
LPS: lipopolysaccharides
FBS: fetal bovine serum
MT: matrine
DEX: dexamethasone
MTT: 3-(4,5-dimethylthiazol-2-yl)-2,5-diphenyltetrazolium bromide
GAPDH: glyceraldehyde-3-phosphate dehydrogenase
TBP: TATA-binding protein
SEM: standard error of the mean
